# The effect of Fe incorporation on the single-crystal elasticity of δ-AlOOH

**DOI:** 10.1007/s00269-025-01319-7

**Published:** 2025-04-18

**Authors:** Niccolò Satta, Giacomo Criniti, Tiziana Boffa Ballaran, Alexander Kurnosov, Takayuki Ishii, Johannes Buchen, Hauke Marquardt

**Affiliations:** 1 Institut für Mineralogie, Universität Münster, 48149 Münster, Germany; 2 Bayerisches Geoinstitut, Universität Bayreuth, 95447 Bayreuth, Germany; 3 Earth and Planets Laboratory, Carnegie Institution for Science, Washington, DC 20015 USA; 4 Institute for Planetary Materials, Okayama University, Misasa, Tottori 682-0193 Japan; 5 Department of Earth Sciences, University of Oxford, Oxford, OX1 3 AN UK

**Keywords:** Elasticity, δ-AlOOH, Water, Anisotropy, Solid solutions, Brillouin scattering

## Abstract

The seismic mapping of hydrous materials in the Earth’s deep interior requires experimental constraints on the elastic anisotropy of hydrous minerals and phases. Oxyhydroxides like δ-(Al,Fe)OOH are arguably the main hosts of water in the lower mantle. Therefore, constraints on the single-crystal elastic tensor of δ-(Al,Fe)OOH solid solutions are crucial to quantify the elastic anisotropy of this material, and advance the current understanding of the recycling of water into the lower mantle. Yet, experimental data for intermediate compositions are scarce, limiting the understanding of how Fe incorporation affects the single-crystal elastic properties of δ-AlOOH. In this study, we provide experimental constraints on the single-crystal elasticity of two δ-(Al,Fe)OOH solid solutions, with Fe/(Al + Fe) of 0.06(1) and 0.133(3). Large single-crystal samples of δ-(Al,Fe)OOH were synthetized at high pressures and temperatures using a multi-anvil press, and the full elastic stiffness tensors were determined at ambient conditions by combining X-ray diffraction and Brillouin scattering measurements. We show that replacing Al^3+^ with Fe^3+^ in δ-(Al,Fe)OOH lowers the magnitude of most coefficients of the elastic stiffness tensor (*c*_ij_), which translates into a substantial reduction of aggregate moduli and acoustic wave velocities. We further show that, at ambient conditions, the acoustic anisotropy of δ-(Al,Fe)OOH displays no sensitivity to Fe–Al substitution.

## Introduction

Water, as hydroxyl groups in minerals, affects the physical, transport and rheological properties, as well as melting regimes of mantle rocks (Hirschmann 2006; Schmidt and Poli 1998). As such, quantifying the deep water recycling, that is the exchange between deep and surficial terrestrial water reservoirs, is crucial to understand the geological evolution of our planet and the development of habitable conditions on Earth (Karato et al. 2020; Peslier et al. 2017).

Water is mainly transferred into the Earth’s mantle by the subduction of altered oceanic lithospheric plates (Hermann and Lakey 2021; Ohtani and Ishii 2024; Walter 2021). Subducting oceanic lithosphere can be broadly divided into three layers according to their distinct petrological features (Ono 1998): (i) a slab mantle, comprising residual of harzburgite and lherzolite, as well as depleted pyrolite (Ringwood and Irifune 1988); (ii) a slab crust, that is basaltic crust sitting above the slab mantle; (iii) a sediment layer, sandwiched between the slab crust and the surrounding Earth’s mantle. Up to relatively shallow upper mantle depths (< 180 km), water is mostly stored in serpentine minerals primarily located along faults forming in the proximity of the subduction trench and capable of penetrating deep into the slab mantle (Faccenda 2014; Faccenda et al. 2008, 2009; Ranero et al. 2003; Ranero and Sallarès, 2004; Ulmer and Trommsdorff 1995). On the other hand, both slab crust and sediments layers are expected to be comparatively dry (Ono 1998). As slabs sink deeper into the transition zone (~ 420–660 km depth), the slab mantle is expected to continue acting as main water reservoir due to the relatively large water storage capability of wadsleyite and ringwoodite (Inoue et al. 1995; Kohlstedt et al. 1996), as well as the formation of dense hydrous magnesium silicates (DHMS) (Angel et al. 2001; Frost 1999; Iwamori 2004; Pamato et al. 2015). However, as slabs penetrate into the lower mantle (~ 660–2890 km), first ringwoodite, and then DHMS (e.g., phase D) break down, possibly leading to the formation of hydrous melts in the uppermost lower mantle (Schmandt et al. 2014) and/or solid solutions of the CaCl_2_-type phase H (MgSiO_4_H_2_), δ-AlOOH, ε-FeOOH and SiO_2_ (Ishii et al. 2022a, b, 2024; Nishi et al. 2019; Ohira et al. 2014; Ohtani 2020; Panero and Caracas 2017). In the latter scenario, the budget of subducted water would be transferred into the slab crust from the slab mantle. These oxyhydroxide solid solutions were found to be stable up to pressure (*P*)-temperature (*T*) conditions of the core-mantle boundary (CMB), enabling water to be delivered and preserved over geological time into the deepest lower mantle (Ohira et al. 2014; Ohtani 2020; Ohtani and Ishii 2024; Yuan et al. 2019).

Geophysical methods like seismic tomography allow for the remote mapping of the Earth’s mantle (e.g., McNamara 2019), from which its thermo-physical state may be inferred (Buchen 2021; Marquardt and Thomson 2020; Thomson et al. 2019; Trautner et al. 2023; Wolf et al. 2015). However, an accurate interpretation of seismological observations requires tight constraints on the elastic properties of mantle constituent minerals. Specifically, the seismic detection of water in the mantle requires tight constraints on the elastic properties of nominally anhydrous and hydrous minerals. These properties have been the focus of numerous experimental studies indeed (Bezacier et al. 2010, 2013; Buchen et al. 2018a, b; Jiang et al. 2006; Rosa et al. 2012, 2015; Sanchez-Valle 2006; Sanchez-Valle et al. 2008; Satta et al. 2019, 2022; Schulze et al. 2018; Su et al. 2021).

Previous experimental studies at lower mantle *P–T* conditions on a Mg-rich MORB + H_2_O reported the formation of H-δ-ε solid solutions, with AlOOH as main molar component and a Fe/(Al + Fe) ratio of ~ 0.05 (Liu et al. 2019), hence making δ-(Al,Fe)OOH particularly relevant for seismic detection of water in the lower mantle. As such, the physical properties of δ-(Al,Fe)OOH have been the target of various recent investigations (Buchen et al. 2021; Insixiengmay and Stixrude 2023; Meier et al. 2022; Sano-Furukawa et al. 2018; Satta et al. 2021, 2024; Strozewski et al. 2023; Thompson et al. 2017, 2020; Trybel et al. 2021). Yet, experimental constraints on the single-crystal elastic properties of δ-(Al,Fe)OOH solid solutions remain extremely poor, as studies are limited to the δ-AlOOH end-member (Wang et al. 2022), and one single intermediate δ-(Al_0.97_Fe_0.03_)OOH composition (Satta et al. 2021, 2024). As such, our understanding of the effect of Fe–Al substitution on the elastic properties of δ-(Al,Fe)OOH is rather limited.

**Fig. 1:**
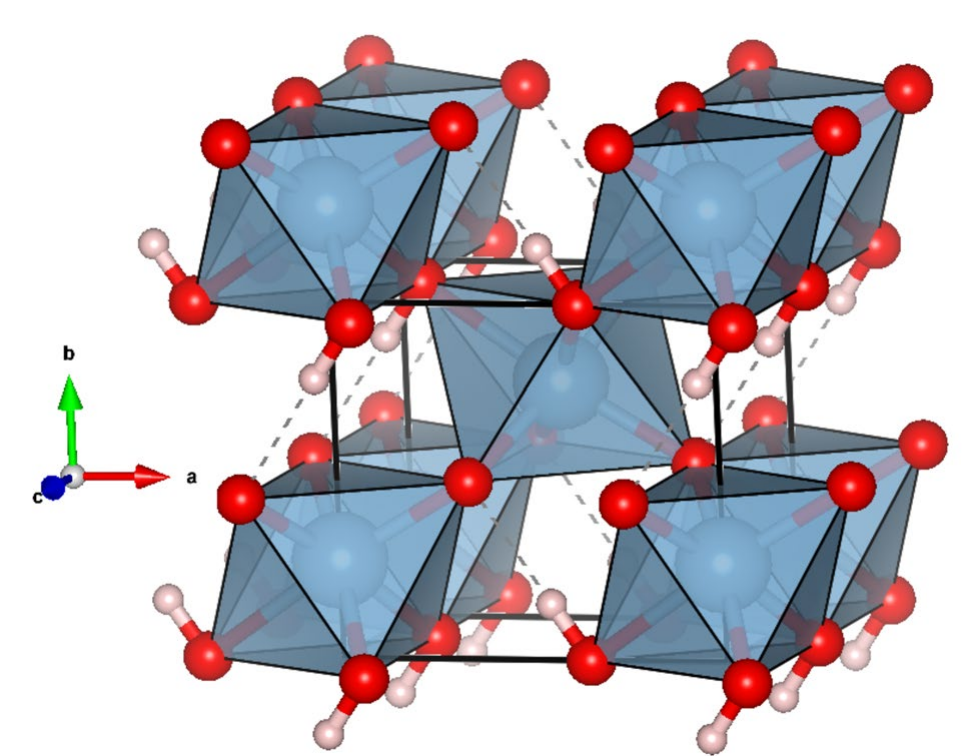
Crystal structure of δ-(Al,Fe)OOH at room conditions (space group *P*2_1_*nm*). Atoms are shown as spheres, with oxygens in red, hydrogens in pink, and (Al,Fe)O_6_ octahedra in blue. Solid lines define the unit-cell, while dashed lines show the position of hydrogen bonds. The drawing was created with VESTA (Momma and Izumi 2011) and using crystal-structure data from Komatsu et al. (2006)

At ambient conditions, δ-(Al,Fe)OOH solid solutions belong to the orthorhombic crystal system (space group *P*2_1_*nm*). Their crystal structure consists of columns of edge-sharing (Al,Fe)O_6_ octahedra running parallel to the *c*-axis and building a framework by sharing vertices (Fig. 1). Cations are octahedrally coordinated by oxygen atoms and hydroxyl groups (OH^–^), with the hydrogen bonds located within the basal plane and pointing inside the channels of the column-based framework (Bolotina et al. 2008; Komatsu et al. 2006; Kuribayashi et al. 2014; Pernet et al. 1975; Suzuki et al. 2000; Suzuki 2010).

Previous compression studies on the *P*2_1_*nm* phase of the Al end-member (δ-AlOOH) constrained its isothermal bulk modulus, *K*_T0_, to 152(2) GPa with a pressure derivative, *K'*_T0_, fixed to 4 (Sano-Furukawa et al. 2009). Similar experimental constraints have also been obtained on intermediate compositions of the δ-(Al_,_Fe)OOH solid solution, such as Fe/(Al + Fe) of ~ 0.03 [*K*_T0_ = 150.4(1.1) GPa; *K'*_T0_ = 3.5(4)], ~ 0.05 [*K*_T0_ = 152(7) GPa, *K'*_T0_ fixed to 4], and ~ 0.12 [*K*_T0_ = 147(1) GPa, *K'*_T0_ fixed to 4] (Ohira et al. 2019; Satta et al. 2024). Interestingly, similar values have been reported for the Fe end-member (ε-FeOOH) as well [*K*_T0_ = 152(4) GPa, *K'*_T0_ = 4.9(2)] (Thompson et al. 2020), hence suggesting that the compressibility of the *P*2_1_*nm* phase of δ-(Al_,_Fe)OOH shows little to no sensitivity to Fe–Al substitution. However, this partially contrasts with ultrasonic measurements on ε-FeOOH showing a significant (about 15%), reduction of the room pressure adiabatic bulk modulus (*K*_S_) with respect to δ-AlOOH (Ikeda et al. 2019; Wang, et al. 2022). More interestingly, expanding this comparison to the aggregate shear modulus (*G*) further highlights a much lower (about 50%) resistance to elastic shear deformation of ε-FeOOH with respect to δ-AlOOH (Ikeda et al. 2019; Wang et al. 2022). Compressional, ${v}_{P}=\sqrt{\frac{{K}_{S}+\frac{3}{4}G}{\rho }}$, and shear,${v}_{S}=\sqrt{\frac{G}{\rho }}$, aggregate velocities are linked to both aggregate moduli and density$\left(\rho \right)$. Due to the relatively large mass of Fe^3+^ compared to Al^3+^, Fe replacing Al should increase the density and hence cause reduction of the aggregate wave velocities of δ-(Al,Fe)OOH. In this regard, Brillouin scattering experiments on powdered δ-(Al,Fe)OOH show that the addition of about 5 mol% of Fe to δ-AlOOH would indeed cause a reduction of ~ 5% in ${v}_{S}$ (Mashino et al. 2016; Su et al. 2021). Yet, aggregate velocities computed from single-crystal data on Fe-bearing δ-(Al_0.97_Fe_0.03_)OOH are surprisingly faster than those observed on powdered δ-AlOOH (Satta et al. 2021, 2024). Such a discrepancy questions our current understanding of the effect of Fe–Al substitution on the elastic properties of δ-(Al,Fe)OOH, hence prompting the need for further experimental constraints.

Here, we describe the single-crystal elastic properties of two δ-(Al,Fe)OOH solid solutions with Fe/(Al + Fe) of 0.06(1) and 0.133(3), determined by a combination of single-crystal X-ray diffraction (XRD) and Brillouin scattering measurements at room conditions. These results allow us to pose tight experimental constraints on the effect of Fe substitution on the single-crystal elastic properties of δ-AlOOH at room conditions.

## Materials and methods

### Synthesis and chemical characterization

**Fig. 2:**
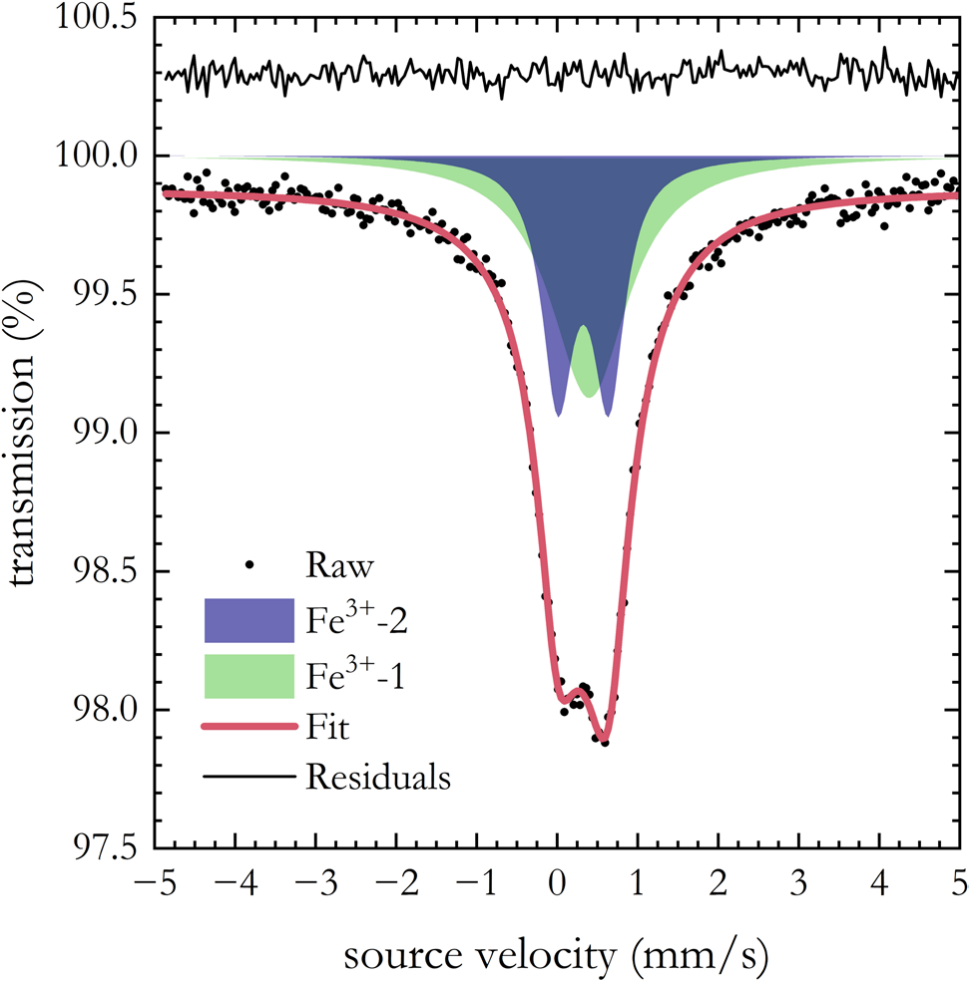
Mössbauer spectrum of Fe6 crystals belonging to the H4802 synthesis run. Spectral components are linked to the quadrupole doublet (Fe^3+^ − 2, in blue) and a superparamagnetic component (Fe^3+^ − 1, in green) of ferric iron (Fe^3+^)

Batches of δ-(Al,Fe)OOH single crystals were synthesized at 21 GPa and 1423 K and 27 GPa and 1423 K in two distinct runs, namely H4802 and I663, respectively. Sample synthesis was performed using the multi-anvil apparatus available at the Bayerisches Geoinstitut (BGI), Universität Bayreuth (Ishii et al. 2016, 2019; Keppler and Frost 2005), and following the procedure delineated by previous studies (Buchen et al. 2021; Kawazoe et al. 2017). In both runs, the starting materials consisted of a finely ground mixture of Al(OH)_3_ and ^57^Fe_2_O_3_ (^57^Fe/ΣFe = 96%), in molar proportion Fe/(Al + Fe) of 0.15 and 0.2 for H4802 and I663 runs, respectively. To determine elemental concentrations, crystals were selected from their respective batch, embedded in epoxy, and analyzed with the JEOL JXA-8100 electron microprobe at the BGI. Al_2_O_3_ corundum and natural almandine were employed as standards for Al and Fe, respectively. On average, the H4802 run (hereafter Fe6) showed Fe/(Al + Fe) equal to 0.06(1) while the I663 run (hereafter Fe13) showed Fe/(Al + Fe) of 0.133(3). The valence state of Fe was determined by Mössbauer spectroscopy, using the constant acceleration spectrometer equipped with a ^57^Co point source installed at the BGI (McCammon, 1994). Measurements were performed on a mosaic of selected Fe6 crystals. A transmission Mössbauer spectrum (Fig. 2) was collected and fitted to a Lorentzian doublet and singlet with the software MossA (Prescher et al. 2012). Since no other contributions to the Mössbauer spectrum were observed, we conclude that 95–100% of the Fe present in the Fe6 samples is in the ferric state state (Fe^3+^). Although Mössbauer spectroscopy measurements were not conducted on Fe13 samples due to the limited number of suitable crystals, we expect all Fe to be in the ferric state as well, based on previous measurements on similar compositions (Kawazoe et al. 2017).

### Sample selection and elasticity experiments

High quality samples were selected based on the sharpness of X-ray diffraction peaks in omega-scan rotations (full width at half maximum < 0.07°). A Huber four-circle Eulerian diffractometer equipped with a Mo-*K*α radiation source (50 kV and 40 mA) and a point detector was operated by SINGLE (Angel and Finger 2011) and employed for sample selection and the determination of unit cell parameters. For each composition, two single-crystal samples were selected, oriented parallel to specific crystallographic planes (*hkl*), and double-side polished down to about 10–15 μm in thickness. Cartesian coordinates (**e**_**1**_**║a, e**_**2**_**║b, e**_**3**_** ║c**) of the platelet normals are (0.62, 0.7, 0.35) for Fe6-X1, (0.18, 0.22, 0.96) for Fe6-X2, (0.12, 0.8, 0.59) for Fe13-X1 and (0.75, 0.01, 0.66) for Fe13-X2. For each composition, unit-cell parameters were determined by centering individually at least 17 and up to 25 reflections in eight equivalent positions (King and Finger 1979) and refined using the vector-least squares method (Ralph and Finger 1982). For Fe6, the unit-cell parameters were found to be equal to *a* = 4.7366(1) Å, *b* = 4.2463(2) Å, *c* = 2.8475(1) Å, and *V* = 57.272(4) Å^3^ while for Fe13 these are *a* = 4.7537(3) Å, *b* = 4.2622(3) Å, *c* = 2.8595(4) Å, and *V* = 57.938(7) Å^3^.

Brillouin scattering measurements were performed at room conditions employing the system available at the BGI (Trots et al. 2011, 2013). Experiments were performed in forward symmetric scattering geometry with an external scattering angle (${\uptheta }ext$) of 80° (Speziale et al. 2014; Whitfield et al. 1976). Given their relatively small size, platelets were loaded in a diamond-anvil cell (DAC) so to ensure scattering geometry preservation during experiments. We note that the DAC served as sample holder only, meaning that the investigated platelets were not pressurized, and that all experiments were conducted at room conditions. A silica reference glass was employed in the calibration of the external scattering angle (${\uptheta }ext$) (Criniti et al. 2021). A Coherent Verdi V2 solid state Nd:YVO_4_ frequency-doubled laser (${\lambda }_{0}$ = 532.0 nm) was focused onto the single-crystal platelets, and a six-pass scanning Fabry-Pérot interferometer (Lindsay et al. 1981; Sandercock 1982) coupled to a single-pixel photon counter and a multi-channel analyzer was used to record Brillouin spectra. Acoustic wave velocities (*v*) were obtained according to the relation (Speziale et al. 2014; Whitfield et al. 1976):

1 $$\begin{array}{*{20}c} {v = \frac{{\Delta \omega \lambda_{0} }}{{2\sin \left( {\frac{{\uptheta ext}}{2}} \right)}}} \\ \end{array}$$

that links the acoustic wave velocities *v* to measured frequency shifts ($\Delta \omega$), incident laser wavelength ($\lambda_{0}$) and external scattering angle ($\uptheta ext$ = 80˚). For each platelet, Brillouin spectra were collected in 10/20° interval step along the 360° azimuth, each orientation corresponding to a different phonon direction.

Given the orthorhombic symmetry of δ-(Al,Fe)OOH, its single-crystal elastic properties are described by a symmetric fourth-rank tensor that has nine independent, non-zero coefficients, namely the elastic stiffness coefficients (*c*_ij_) that in Voigt notation are: *c*_11_, *c*_22_, *c*_33_, *c*_44_, *c*_55_, *c*_66_, *c*_12_, *c*_13_, *c*_23_ (Nye 1985). The full elastic stiffness tensor was obtained by inverting experimental data in a least-square fit of the Christoffel equation (Haussühl 2007):

2 $$\begin{array}{*{20}c} {\left| {c_{ijkl} n_{j} n_{l} - \rho v^{2} \delta_{ik} } \right| = 0 } \\ \end{array}$$

where *c*_*ijkl*_ are the elastic stiffness coefficients in full tensor notation, *n*_*j*_ and* n*_*l*_ are the direction cosines defining the propagation direction of acoustic waves, and *ρ* and *δ*_*i*k_ are density and the Kronecker delta, respectively. The least-square fitting of the Christoffel equation was run in Origin (OriginLab corporation, Northampton, MA, USA) following previous formulations (Buchen 2018), and correcting for platelet tilting when necessary (Kurnosov et al. 2017). Densities were calculated using both unit-cell dimensions and compositions determined in this study, and accounting for the enrichment in ^57^Fe. Voigt and Reuss bounds of the adiabatic bulk (*K*_S_) and shear moduli (*G*) were calculated using the *c*_ij_ and the elastic compliance coefficients, *s*_ij_ (i.e., = *c*_ij_^–1^, in matrix notation), respectively*.* Hill averaged values for each aggregate moduli were obtained by taking the mean of the Reuss and Voigt values (Hill 1952). All results are reported in Table 1.

**Table 1:** Elastic properties of δ-(Al,Fe)OOH at room conditions with varying Fe content

	Wang et al. (2022)	Satta et al. (2024)	Fe6	Fe13
Fe/(Fe + Al)	0	0.026(1)	0.06(1)	0.133(3)
ρ (g/cm^3^)	3.536(1)	3.559(2)	3.58(2)	3.66(2)
*β*_a_ (10^3^/GPa)	*1.91(6)*	*2.05(2)*	*1.94(4)*	*2.31(1)*
*β*_b_ (10^3^/GPa)	*2.78(2)*	*2.79(2)*	*2.83(4)*	*3.09(2)*
*β*_c_ (10^3^/GPa)	*1.56(1)*	*1.53(1)*	*1.56(5)*	*1.46(1)*
*c*_11_ (GPa)	375.9(9)	360(2)	358(2)	329(1)
*c*_22_ (GPa)	295.4(11)	295(2)	285(1)	270(2)
*c*_33_ (GPa)	433.5(12)	414(2)	404(4)	397(1)
*c*_44_ (GPa)	129.2(6)	126.6(6)	121(2)	117(1)
*c*_55_ (GPa)	133.4(7)	126.8(4)	123(3)	116.4(3)
*c*_66_ (GPa)	166.4(6)	168.2(9)	158(1)	150(1)
*c*_12_ (GPa)	49.7(9)	41.0(24)	52(1)	31(1)
*c*_13_ (GPa)	91.9(15)	96.0(16)	101(6)	98(1)
*c*_23_ (GPa)	52.8(21)	61(2)	61(5)	62(1)
$K^{VRH}$ (GPa)	162.9(31)	159.8(9)	161.0(22)	150(1)
$G^{VRH}$ (GPa)	145.2(13)	141.1(3)	134.7(8)	129.0(3)
*v*_P_ (km/s)	10.04(7)	9.89(1)	9.75(4)	9.37(3)
*v*_S_ (km/s)	6.41(3)	6.30(1)	6.13(3)	5.94(2)

Values in italics are determined from their respective *c*_ij_ dataset. Values in parenthesis are uncertainties on the last digit

## Results and discussion

**Fig. 3:**
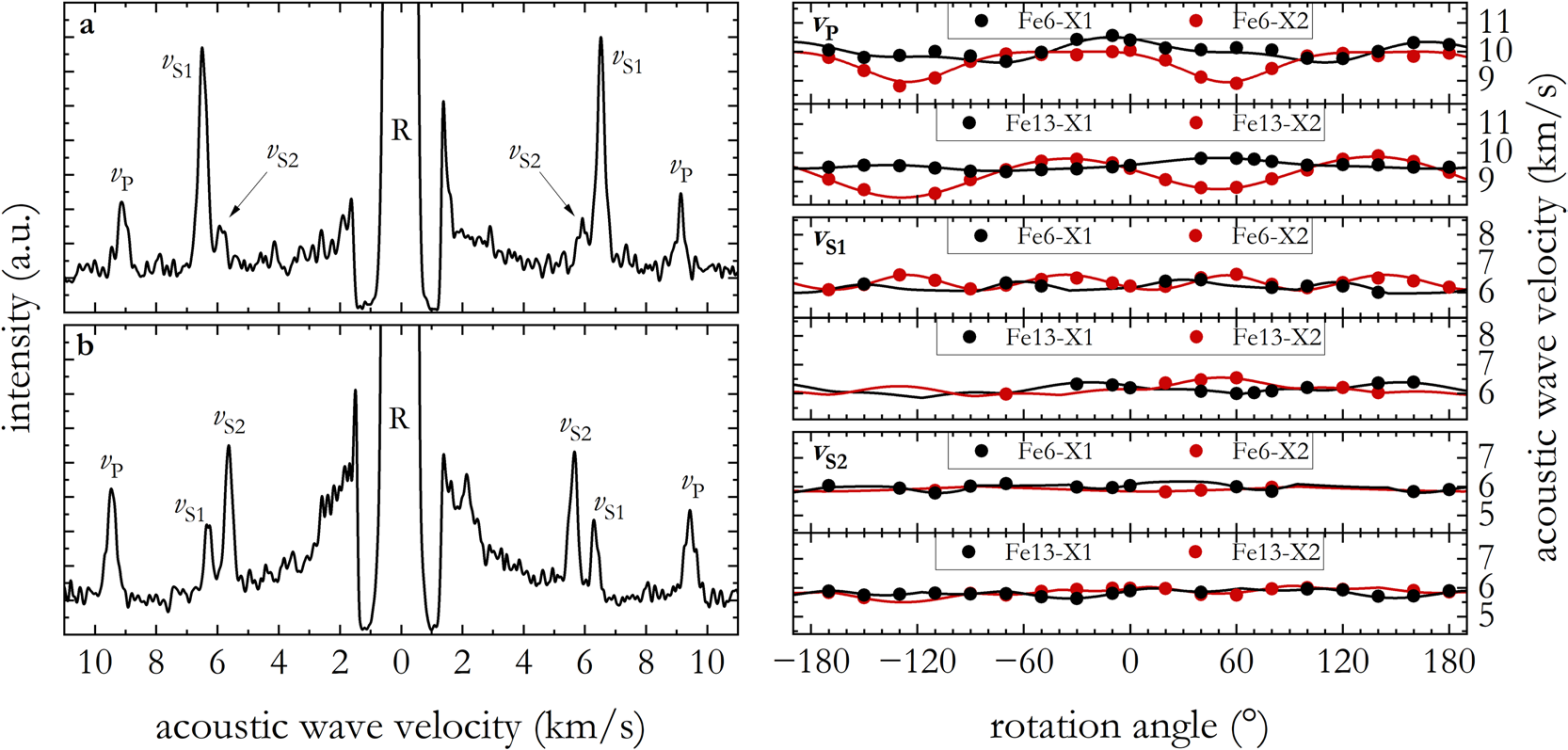
Results from Brillouin scattering experiments conducted on δ-(Al,Fe)OOH at ambient conditions. In (**a**, **b**), representative Brillouin spectra collected on Fe6 and Fe13 samples, respectively, are shown; spectral features belong to the sample compressional (*v*_P_), fast shear (*v*_S1_) and slow shear (*v*_S2_) wave velocities, and elastic peak (R). Features in the low-velocity range (< 3 km/s) are unattenuated tails of the elastic peak. The plots on the right show the experimental angular dispersion of compressional (*v*_P_)*,* fast shear (*v*_S1_), and slow shear (*v*_S2_) acoustic wave velocities as a function of the rotation angle. Measured values are plotted as solid symbols, while solid curves are calculated from the best-fit *c*_ij_ determined from experimental data inversion (see “Materials and methods” section)

Two representative Brillouin spectra, one for each of the two compositions studied here, are shown in Fig. 3a. Figure 3b shows acoustic wave velocities obtained via Brillouin scattering experiments together with those calculated from the best-fit *c*_ij_ determined from an inversion of experimental data.

### Single-crystal elasticity

Our results show that the *c*_ij_ display the systematics observed on less Fe-rich compositions (Satta et al. 2024; Wang et al. 2022). Specifically, the relations between the diagonal and off-diagonal coefficients are *c*_33_ > *c*_11_ > *c*_22_ and *c*_13_ > *c*_23_ > *c*_12_, respectively. For an orthorhombic crystal, axial compressibility can be determined from the diagonal and off-diagonal compliance coefficients *s*_ij_ according to the relation (Nye 1985):

3 $$\begin{array}{*{20}c} {\beta_{i} = s_{i1} + s_{i2} + s_{i3} } \\ \end{array}$$

with* i* being 1, 2 and 3 for the *a*-, *b*- and *c*-axis, respectively. At room conditions and for both compositions, we found the *c*-axis to be the least compressible, while the *b*-axis shows the largest compressibility, consistent with previous compressional studies (Kuribayashi et al. 2014; Ohira et al. 2019; Sano-Furukawa et al. 2009).

We found the diagonal shear *c*_ij_ coefficients (i = j = 4, 5, 6 in Voigt notation) to follow the scheme *c*_66_ > *c*_55_ ≈ *c*_44_, in agreement with previous studies on δ-(Al_0.97_Fe_0.03_)OOH (Satta et al. 2024). On the other hand, previous Brillouin experiments on δ-AlOOH single crystals have found the relationship among shear coefficients *c*_ij_ to be *c*_66_ > *c*_55_ > *c*_44_, although it is important to note that the difference between *c*_55_ and *c*_44_ was only 3% (Wang et al. 2022).

**Fig. 4:**
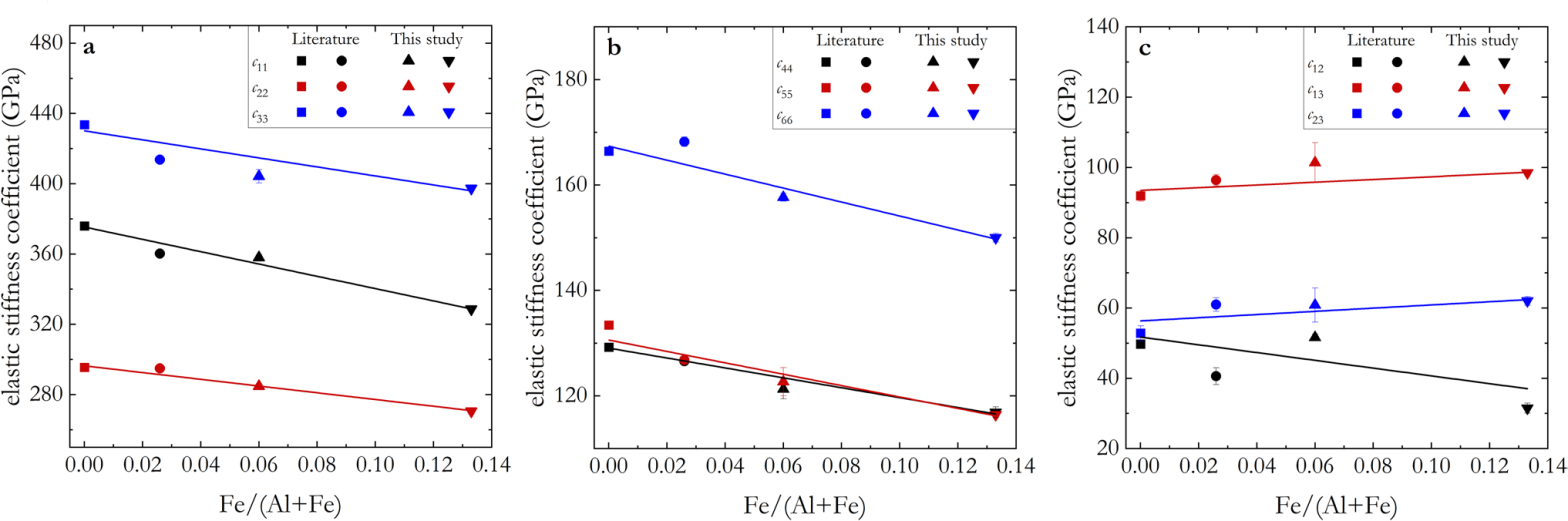
Elastic stiffness coefficients (*c*_ij_) of δ-(Al,Fe)OOH single crystals at room conditions plotted as a function of the Fe/(Al + Fe) molar ratio. **a** *c*_11_,* c*_22_ and *c*_33_; **b** *c*_44_, *c*_55_ and *c*_66_; **c** *c*_12_, *c*_13_ and *c*_23_. Solid symbols are experimentally determined values, while solid lines result from linear fits to the experimental values. Literature data for Fe0 (solid squares) and Fe3 (solid circles) are from Wang et al. (2022) and Satta et al. (2024), respectively

The effect of Fe–Al substitution on the single-crystal elastic properties of δ-(Al,Fe)OOH at room conditions is displayed by plotting *c*_ij_ as a function of Fe/(Al + Fe) molar ratio in Fig. 4. In this study, only experimentally determined single-crystal elasticity data were taken into consideration for the comparison, as computational studies only focused on the AlOOH end member and the effect of pressure (Cortona 2017; Pillai et al. 2018; Tsuchiya and Tsuchiya 2009).

Within the investigated compositional range, we found Fe substituting Al to influence the *c*_ij_ linearly as described by the following equations (yielding *c*_ij_ values in GPa):

4 $$c_{11} = 375\left( 2 \right) - 351\left( {23} \right) \cdot {\text{Fe}}/\left( {{\text{Al}} + {\text{Fe}}} \right)$$

5 $$c_{22} = 296\left( 1 \right) - 191\left( {21} \right) \cdot {\text{Fe}}/\left( {{\text{Al}} + {\text{Fe}}} \right)$$

6 $$c_{33} = 430\left( 5 \right) - 257\left( {58} \right) \cdot {\text{Fe}}/\left( {{\text{Al}} + {\text{Fe}}} \right)$$

7 $$c_{44} = 129.1\left( 3 \right) - 94\left( 8 \right) \cdot {\text{Fe}}/\left( {{\text{Al}} + {\text{Fe}}} \right)$$

8 $$c_{55} = 131\left( 1 \right) - 108\left( {14} \right) \cdot {\text{Fe}}/\left( {{\text{Al}} + {\text{Fe}}} \right)$$

9 $$c_{66} = 167\left( 2 \right) - 132\left( {30} \right) \cdot {\text{Fe}}/\left( {{\text{Al}} + {\text{Fe}}} \right)$$

10 $$c_{12} = 52\left( 5 \right) - 110\left( {75} \right) \cdot {\text{Fe}}/\left( {{\text{Al}} + {\text{Fe}}} \right)$$

11 $$c_{13} = 93\left( 2 \right) + 39\left( {15} \right) \cdot {\text{Fe}}/\left( {{\text{Al}} + {\text{Fe}}} \right)$$

12 $$c_{23} = 56\left( 3 \right) + 45\left( {28} \right) \cdot {\text{Fe}}/\left( {{\text{Al}} + {\text{Fe}}} \right)$$

with Fe/(Fe + Al) being the molar ratio. Both intercept and slope values were simultaneously refined, taking into account* c*_ij_ uncertainties, in a weighted least-square fitting procedure. All *c*_ij_, but *c*_13_ and *c*_23_, are highly sensitive to Fe–Al substitution and display a significant reduction as Fe is incorporated into δ-(Al,Fe)OOH. It is worth noting, however, that extrapolating our fitting results toward increasing Fe content and ultimately to the Fe end member would lead to an almost complete suppression of the magnitude of $c_{11}$. To our knowledge, no experimental determination of the elastic tensors of ε-FeOOH has been reported so far, preventing us to further elaborate on a possible non-linearity. Therefore, it is important to stress that the linear approximation proposed in this study holds valid only up to the highest Fe content investigated here.

In octahedral (6-fold) coordination, the ionic radius of Fe^3+^ is slightly larger than that of Al^3+^ (Shannon 1976). Hence, Fe^3+^ replacing Al^3+^ in δ-(Al,Fe)OOH decreases the stiffness of octahedra and the overall resistance to elastic shear deformation of the crystal structure. Additionally, as the octahedral volume increases, the inter-octahedral O–O distances also increase, thus weakening hydrogen bonds (Kollman and Allen 1972). This could provide a reasonable explanation—at least to a first-approximation—for the behavior exhibited by most *c*_ij_ with increasing Fe content. On the other hand, the low-sensitivity of *c*_13_ and *c*_23_ to Fe–Al substitution may suggest a more complex mechanism with which Fe–Al substitution influences the elastic anisotropy of δ-(Al,Fe)OOH. Additional experimental investigations aiming, for example, at quantifying the relation between physical properties and crystal structure will be needed to further advance our understanding of the behavior of off-diagonal coefficients *c*_ij_ in δ-(Al,Fe)OOH.

### Acoustic anisotropy

Our experimental results allow the calculation of acoustic wave velocities along any given crystallographic direction, thus permitting the evaluation of the anisotropy of acoustic wave velocities. Here, we focused on identifying the largest difference in percentage between the fastest and slowest compressional wave velocities, that are $v_{{\text{P}}}^{{{\text{MAX}}}}$ and $v_{{\text{P}}}^{{{\text{MIN}}}}$, respectively. This was done by computing the $v_{P}$ the azimuthal anisotropy ($Av_{{\text{P}}}$):

13 $$\begin{array}{*{20}c} {Av_{{\text{P}}} = \frac{{\left( {v_{{\text{P}}}^{{{\text{MAX}}}} - v_{{\text{P}}}^{{{\text{MIN}}}} } \right)}}{{\left( {v_{{\text{P}}}^{{{\text{MAX}}}} + v_{{\text{P}}}^{{{\text{MIN}}}} } \right)}} \cdot 200\left( \% \right)} \\ \end{array}$$

Additionally, we determined the largest difference (in percentage) between fast and slow shear waves propagating along a specific direction, that is the $v_{{\text{S}}}$ radial anisotropy (or shear-wave splitting factor):

14 $$\begin{array}{*{20}c} {Av_{{\text{S}}} = \frac{{\left( {v_{{{\text{S}}1}} - v_{{{\text{S}}2}} } \right)}}{{\left( {v_{{{\text{S}}1}} + v_{{{\text{S}}2}} } \right)}} \cdot 200\left( \% \right)} \\ \end{array}$$

δ-AlOOH shows the highest $Av_{{\text{S}}}$ of 12.65% along the [010], and an $Av_{P}$ of 19.1%, with $v_{P}^{MAX}$ and $v_{P}^{MIN}$ along the [001] and [010], respectively (Wang et al. 2022). These values are in good agreement with our results in terms of orientation and magnitude. Specifically, we determined $Av_{P}$ in Fe6 to be equal to 17(2)%, and to 19(1)% in Fe13, with $v_{{\text{P}}}^{{{\text{MAX}}}}$ and $v_{{\text{P}}}^{{{\text{MIN}}}}$ along the [001] and [010], respectively. As for $Av_{{\text{S}}}$, this was found to reach its largest magnitude of 13(1)% along [010] in both Fe6 and Fe13. Therefore, the incorporation of Fe into the crystal structure of δ-(Al,Fe)OOH seems to have no significant effect on the acoustic anisotropy at ambient conditions, at least up to the highest Fe/(Al + Fe) investigated here.

According to the convention (**e**_**1**_**║a, e**_**2**_**║b, e**_**3**_**║c**), the [001] direction in Cartesian coordinates is oriented parallel to the *c*-axis of the crystallographic reference system. Since the $v_{{\text{P}}}^{{{\text{MAX}}}}$ is found along the [001], it will be equal to $\sqrt {\frac{{c_{33} }}{\rho }}$, while $v_{{\text{P}}}^{{{\text{MIN}}}}$ is equal to $\sqrt {\frac{{c_{22} }}{\rho }}$ as it propagates along the [010]. Therefore, by interpreting $Av_{{\text{P}}}$ in terms of structural features of δ-(Al,Fe)OOH, we see that the chains of edge-sharing octahedra running along the *c*-axis form the stiffest structural unit. As a result, compressional waves that propagate along the *c*-axis are the fastest while corner-sharing connections between octahedral chains cause the [010] direction to be relatively more compressible (*c*_22_ < *c*_33_) and result in compressional wave velocities propagating along this direction to be the slowest.

Focusing on $Av_{{\text{S}}}$, we see that the maximum value is observed for shear waves propagating along [010] for both Fe6 and Fe13. Along the [010] direction, the fast shear wave ${ }v_{{{\text{S}}1}}$ is polarized in the (001) plane with a velocity equal to $\sqrt {\frac{{c_{66} }}{\rho }}$. On the other hand, the slow shear wave $v_{{{\text{S}}2}}$ is polarized in the (100) plane and propagates along [010] with a velocity of $\sqrt {\frac{{c_{44} }}{\rho }}$. Therefore, the $Av_{S}$ in δ-(Al,Fe)OOH is strictly controlled by the contrast in terms of resistance to shear in the (100) and (001) planes. It is important to note, however, that our results show *c*_44_ to be equal to *c*_55_ within uncertainties. Therefore, the resistances to shear deformation in the (100) and (010) planes are equal within uncertainties. As a result, $Av_{{\text{S}}}$ values along the [010] and [100] are equal within uncertainties in both Fe6 and Fe13.

According to our results, Fe–Al substitution causes a notable reduction of acoustic wave velocities, including along those directions where the largest acoustic anisotropy has been determined. Yet, a comparison of our study with previous experimental results clearly shows that the acoustic anisotropy is not sensitive to the Fe–Al substitution. This can be explained by considering the *c*_ij_ controlling acoustic anisotropy, and specifically their evolution with varying Fe content up to Fe/(Al + Fe) = 0.133(3) (Eqs. 4–12). The compressional wave anisotropy is primarily governed by *c*_22_ and *c*_33_. These two elastic stiffness coefficients have comparable slopes in the *c*_ij_-Fe/(Al + Fe) space, leading the longitudinal elastic anisotropy *c*_33_/*c*_22_ to remain relatively constant with increasing Fe content, and in turn causing $Av_{P}$ to exhibit a limited sensitivity to Fe–Al substitution. Similarly, the shear elastic anisotropy* c*_66_*/c*_44_ is expected to remain relatively constant with increasing Fe content, due to the comparable slopes of these two shear coefficients *c*_ij_. As a result, $Av_{S}$ exhibits little to no sensitivity to Fe–Al substitution. We further point out that *c*_44_ and* c*_55_ exhibit very similar slopes, too. Therefore, within the investigated compositional range, we expect $Av_{S}$ to display a low sensitivity to Fe–Al substitution for propagation along [100] (hence linked to *c*_66_/*c*_55_) as well. At high pressures, however, the structural phase transition and changes in hydrogen bond configuration around 8 GPa may impact anisotropy (Satta et al. 2024).

### Aggregate properties

**Fig. 5:**
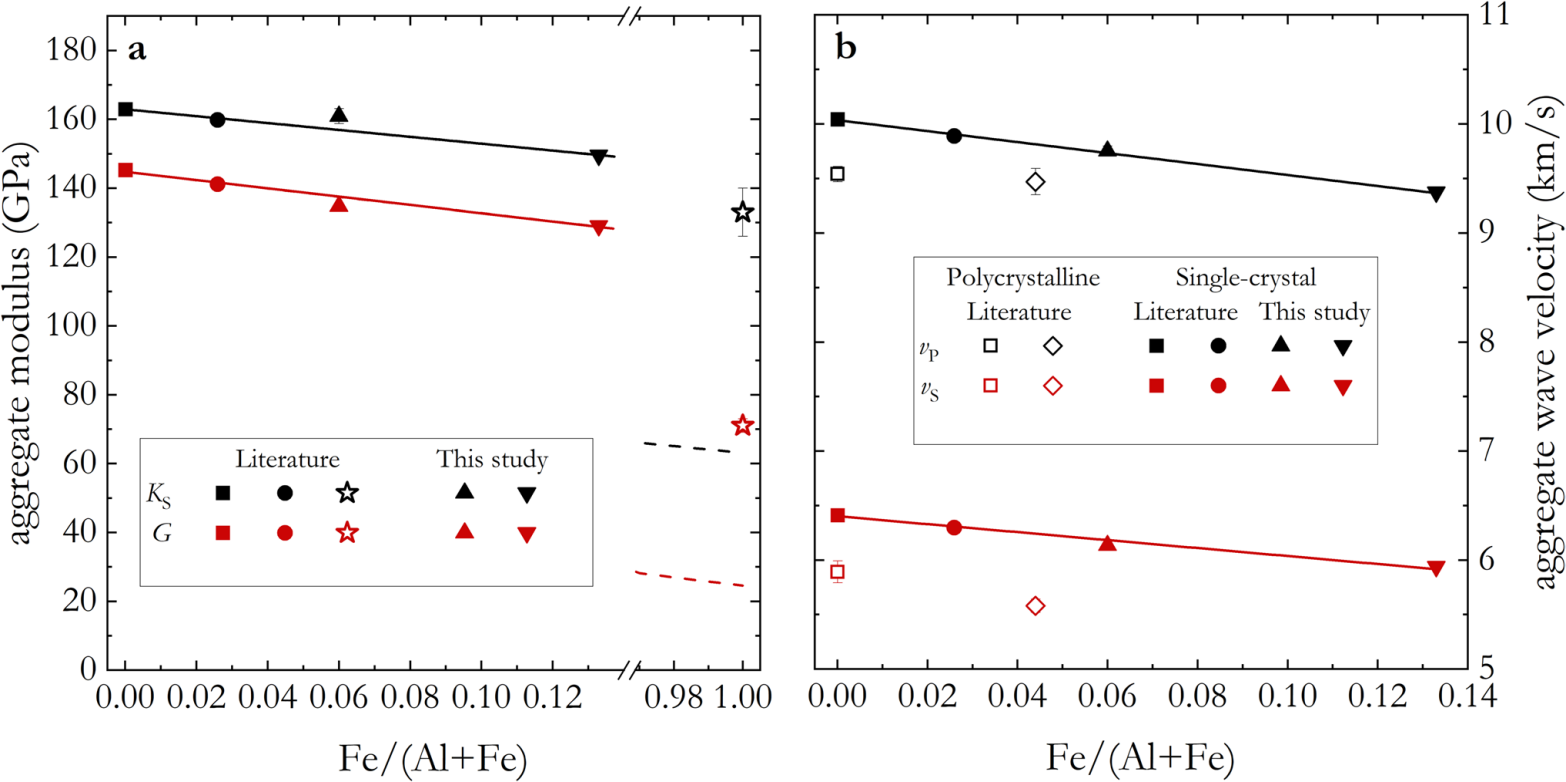
Aggregate properties of δ-(Al,Fe)OOH at room conditions plotted as a function of the Fe/(Al + Fe) molar ratio. Adiabatic aggregate moduli and velocities are shown in (**a**, **b**), respectively. In (**a**), literature data are shown as solid squares, and circles are results from Wang et al. (2022) and Satta et al. (2024), respectively. Open stars are the results for ε-FeOOH by Ikeda et al. (2019) and were not considered in the fit. Solid lines are linear fits to observations within the investigated compositional range, while dashed lines extrapolate linear fits toward higher Fe contents. In (**b**), solid symbols are values determined from single-crystal experimental data, with solid squares and circles used to plot literature results from Wang et al. (2022) and Satta et al. (2024), respectively. Open symbols are values from Brillouin experiments on polycrystalline samples, with squares and diamonds showing the results by Mashino et al. (2016) and Su et al. (2021) respectively

Aggregate properties determined in this study at room condition are listed in Table 1, and plotted against Fe/(Al + Fe) molar ratio in Fig. 5. In Fig. 5a, our results on the adiabatic bulk, *K*_S_, and shear, *G*, moduli of δ-(Al,Fe)OOH are plotted together with those obtained from single-crystal experimental data available in the literature (Satta et al. 2024; Wang et al. 2022). The effect of Fe–Al substitution on the aggregate moduli up to Fe/(Al + Fe) = 0.133(3) is significant, and can be parametrized through the following equations (yielding aggregate moduli values in GPa):

15 $$\begin{array}{*{20}c} {K_{S} = 163\left( 1 \right) - 100\left( 9 \right) \cdot {\text{Fe}}/\left( {{\text{Al}} + {\text{Fe}}} \right)} \\ \end{array}$$

16 $$\begin{array}{*{20}c} {G = 144.7\left( 7 \right) - 120\left( 9 \right) \cdot {\text{Fe}}/\left( {{\text{Al}} + {\text{Fe}}} \right)} \\ \end{array}$$

with Fe/(Al + Fe) being the molar ratio. Both intercept and slope values were refined, taking into account elastic moduli uncertainties, in a weighted least-square fitting routine. We note, however, that extrapolating our fit toward higher Fe content (dashed lines in Fig. 5a) results in an underestimation of the ε-FeOOH aggregate moduli, as shown through comparison with experimental data by previous studies (Ikeda et al. 2019). This is a direct result of the behavior exhibited by the *c*_ij_ discussed above. Unfortunately, a lack of data for more Fe-rich compositions does not allow us to further elaborate on a possible non-linear behavior of the elastic moduli as a function of Fe–Al substitution. Therefore, we reiterate that the linear approximation proposed in this study holds valid only up to the highest Fe content investigated here.

In Fig. 5b, single-crystal data are plotted together with results from Brillouin scattering data on polycrystalline samples of δ-(Al,Fe)OOH (Mashino et al. 2016; Su et al. 2021). Both aggregate velocities decrease with increasing iron content due to the relation between aggregate velocities, density, and moduli, in a behavior that is described by the following equations (yielding aggregate velocity values in km/s):

17 $$\begin{array}{*{20}c} {v_{P} = 10.03 - 5.0\left( 2 \right) \cdot {\text{Fe}}/\left( {{\text{Al}} + {\text{Fe}}} \right)} \\ \end{array}$$

18 $$\begin{array}{*{20}c} {v_{S} = 6.4 - 3.7\left( 3 \right) \cdot {\text{Fe}}/\left( {{\text{Al}} + {\text{Fe}}} \right)} \\ \end{array}$$

with Fe/(Al + Fe) being the molar ratio. Intercept and slope values were obtained, taking into account aggregate velocity uncertainties, through a weighted least-square fitting. Interestingly, we notice that aggregate velocities determined on polycrystalline samples do not follow the linear trend delineated by single-crystal data. Specifically, polycrystalline δ-AlOOH exhibits velocities comparable to those of our Fe-richest composition (Fe13) while δ-(Al_0.956_Fe_0.044_)OOH, and particularly its *v*_S_, exhibits about 10% slower velocities than those determined from Fe6 single-crystal data, despite the comparable composition. A similar mismatch between sound wave velocities determined on single crystals and polycrystalline samples has been observed for MgSiO_3_ bridgmanite (Criniti et al. 2021; Murakami et al. 2007). Here we show that Fe incorporation in δ-AlOOH translates into a reduction of aggregate velocities, hence strengthening previous suggestions that such a mismatch between polycrystalline and single-crystal data of δ-(Al,Fe)OOH cannot be ascribed to the Fe–Al substitution (Satta et al. 2024). Brillouin scattering measurements on polycrystalline samples may be affected by the development of a preferred orientation in the sample, but also by selective elasto-optic coupling (Speziale et al. 2014), grain-boundary properties (Marquardt et al. 2011), and grain-grain interactions (Buchen et al. 2018a, b; Wang et al. 2023), all of which may contribute to the mismatch observed in the comparison between polycrystalline and single-crystal data of δ-(Al,Fe)OOH.

## Conclusions

In this study, the single-crystal elastic properties of two δ-(Al,Fe)OOH solid solutions with Fe/(Al + Fe) of 0.06(1) and 0.133(3) have been investigated by combining single-crystal X-ray diffraction and Brillouin scattering measurements at ambient conditions.

We found that the off-diagonal *c*_ij_, as well as both compressional and shear *c*_ij_ of the here-studied δ-(Al,Fe)OOH solid solutions display the same systematic as for δ-AlOOH (Wang et al. 2022). Additionally, we show that the incorporation of Fe^3+^ at the expense of Al^3+^ in δ-AlOOH causes a linear reduction of the magnitude of most c_ij_ up to the highest Fe content investigated in this study [Fe/(Al + Fe) = 0.133(3)].

In terms of acoustic anisotropy, we identified chains of edge-sharing octahedra parallel to the *c*-axis to play a key role in $Av_{{\text{P}}}$, while $Av_{{\text{S}}}$ is governed by different shear resistances in the (100) and (001) planes. Up to the highest Fe content here-investigated, we found that the acoustic anisotropy remains constant as Fe replaces Al in δ-(Al,Fe)OOH, which reflects the negligible sensitivity of elastic anisotropy to this cation substitution.

We found the behavior exhibited by the c_ij_ as a function of Fe/(Al + Fe) molar ratio to be reflected on both aggregate *K*_S_ and *G* elastic moduli δ-(Al,Fe)OOH. As such, Fe incorporation causes a decrease in terms of magnitude of both aggregate moduli, which can be approximated to be linear up to the highest Fe content investigated in this study. A similar behavior is displayed by $v_{{\text{P}}}$ and $v_{{\text{S}}}$ aggregate velocities due to their relationship with aggregate moduli and density.

## Data Availability

Data is provided within the manuscript.
